# Efficacy and clinical and neuroimaging biomarkers of response of two intensive and spaced treatment protocols of theta burst magnetic stimulation in treatment resistant depression

**DOI:** 10.1192/j.eurpsy.2023.1269

**Published:** 2023-07-19

**Authors:** Y. Cañada, P. Benavent, A. Sabater, P. Navalón, M. Beser, L. Martí-Bonmatí, P. Sierra

**Affiliations:** 1 Mental Health Group, La Fe Health Research Institute; 2 Psychiatry, La Fe University and Polytechnic Hospital; 3 GIBI, La Fe Health Research Institute, Valencia, Spain

## Abstract

**Introduction:**

Resistant to treatment depression (RTD) is a prevalent disease that implies functional impairment and high resources consumption. Theta Burst Transcranial Magnetic Stimulation (TBS) in dorsolateral prefrontal cortex (DLPFC) is a novel therapy that has shown experimental efficacy and as an adjuvant strategy in RTD. The implementation of TBS in the Public National Health Service requires cost-effective protocols that achieve earlier responses and higher rates of effectiveness, and whose design is based on biomarkers of response so as to adequately select candidate patients.

**Objectives:**

To assess the efficacy and safety of novel bilateral and unilateral intensive/spaced protocols of TBS in outpatients with unipolar/bipolar RTD compared with sham stimulation. Specific objectives: I) Comparison of mood change at the end of TBS protocol in the groups and maintenance of its effect at 3 months; II) Characterization of cerebral connectivity and metabolism patterns related to the effects of TBS; III) Analysis of the interaction between clinical and neuroimaging predictors so as to determine a RTD profile of patient that can benefit from TBS.

**Methods:**

A two-year randomized double-blind clinical trial with 96 outpatients with TRD will be carried out. Participants will be randomized in three groups (active bilateral, active left and sham right and sham bilateral) to receive 22 active/sham sessions of continuous TBS (right DLPFC) and intermittent TBS (left DLPFC) during 6 weeks (w 1-2: 5 sessions/w, w 3-6: 3 sessions/w). Assessments of mood and side-effects will be carried out weekly. Functional neuroimaging will be a a simultaneous PET/MR acquisition previous and at the end of TBS treatment. Between-group comparisons of efficacy in terms of Hamilton Depression Rating Scale (HDRS-17) from basal to 6th week will be performed using controlled mixed regression models. Between-group comparisons will be made at baseline and after treatment, studying the imaging biomarkers obtained. Clinical and neuroimaging predictors of response will be integrated in machine learning models.

**Results:**

The expected results of the project are summarized in the following hypotheses: 1) The intensive and spaced protocols of TBS as an adjuvant antidepressant treatment will have greater efficacy than sham stimulation in patients with TRD. 2) Both protocols will be safe, with mild side effects. 3) Unilateral and bilateral TBS protocols will involve changes in connectivity and cerebral metabolic consumption mainly in regions of the fronto-cingulo-temporal circuitry. 4) PET/MR imaging biomarkers will allow us to differentiate whether patients have responded to treatment with TBS.

**Conclusions:**

This project may help to improve resistant to treatment depression management by personalizing TMS treatment with the use of neuroimaging biomarkers.

**Disclosure of Interest:**

None Declared

